# P-1090. Rifaquizinone for the Treatment of Acute Bacterial Skin and Skin Structure Infections: A Multicenter, Double-Blind, Randomized, Active-Controlled, Phase 2 Trial

**DOI:** 10.1093/ofid/ofae631.1278

**Published:** 2025-01-29

**Authors:** J Scott Overcash, Michael Waters, Huan Wang, Guozhu Geng, Changlin Ai, Zhenkun Ma, Jing Chen

**Affiliations:** Velocity Clinical Research, San Diego, California; Velocity Clinical Research, San Diego, California; TenNor Therapeutics (Suzhou) Ltd, Suzhou, Jiangsu, China (People's Republic); TenNor Therapeutics (Suzhou) Ltd, Suzhou, Jiangsu, China (People's Republic); TenNor Therapeutics (Suzhou) Ltd, Suzhou, Jiangsu, China (People's Republic); TenNor Therapeutics, Suzhou Industrial Park, Jiangsu, China (People's Republic); TenNor Therapeutics (Suzhou) Ltd, Suzhou, Jiangsu, China (People's Republic)

## Abstract

**Background:**

Rifaquizinone (RFQ, TNP-2092) is under development for the treatment of serious or life-threatening bacterial infections including those caused by gram-positive pathogens that have developed resistance to commonly used antibiotics. This study compared the safety, tolerability and efficacy of RFQ and vancomycin in patients with acute bacterial skin and skin structure infections (ABSSSIs).

**Figure:**
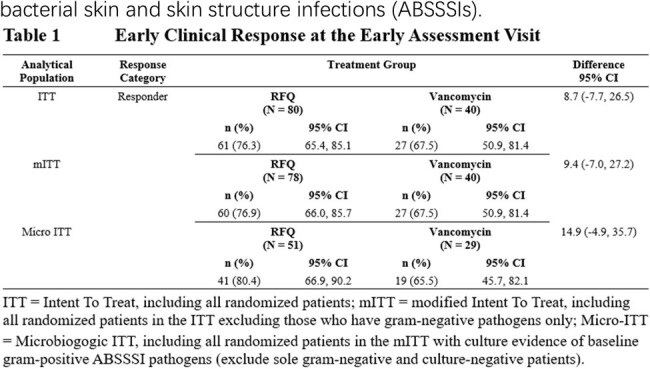

**Methods:**

A multi-center, randomized, double blind, positive controlled phase 2 clinical trial (NCT03964493) was conducted in adults with ABSSSIs in the USA. Eligible patients were randomized 2:1 to receive RFQ (300 mg) or vancomycin (1 g) IV q12h for 3 to 14 days. Patients who met the switching criteria may switch to commercially available oral antibiotics judged by the investigators. The total duration of the treatment was 7 to 14 days. The primary endpoint was safety and tolerability. The key secondary endpoint was the clinical response at early assessment (EA) visit.

**Results:**

A total of 117 patients received at least 1 dose of study drugs. The incidences of adverse events (AEs) in RFQ and vancomycin groups were 46.2% (36/78) and 48.7% (19/39), respectively. The most of AEs were mild in severity. No drug-related serious AE was reported. The clinical response rate of RFQ at EA visit was higher than vancomycin in the ITT, modified ITT and Micro-ITT populations (Table 1). In patients infected with MRSA and fluoroquinolone-resistance (RF-Q) *S. aureus*, the clinical response rate of RFQ and vancomycin group were 78.1% (25/51) *vs* 57.9% (11/29), and 75.9% (22/51) *vs* 55.6% (10/29), respectively. Pharmacokinetic-pharmacodynamic (PK-PD) analysis indicated that more than 95% of patients achieved the systemic exposures required (AUC_0-24h_ > 50 µg·h/mL) for the treatment of medical device associated biofilm infections.

**Conclusion:**

RFQ was safe and well tolerated in patients with ABSSSIs. The early clinical response rates of RFQ in ITT, mITT and micro-ITT populations were all higher than that of vancomycin. More importantly, RFQ demonstrated higher early clinical response rates than vancomycin in patients infected with MRSA and FQ-R *S. aureus*. The PK-PD analysis indicated that sufficient drug exposures were achieved for medical device associated biofilm infections.

**Disclosures:**

**Huan Wang, PhD**, TenNor Therapeutics (Suzhou) Ltd: Employee **Guozhu Geng, MD**, TenNor Therapeutics (Suzhou) Ltd: Employee **Changlin Ai, Master of Medicine**, TenNor Therapeutics (Suzhou) Ltd: Employee **Zhenkun Ma, PhD**, TenNor Therapeutics (Suzhou) Ltd: Employee **Jing Chen, Master of Science**, TenNor Therapeutics (Suzhou) Ltd: Employee

